# Systemic Analyses of Anti-Cell-Senescence Active Compounds in *Camellia* Sect. *Chrysantha* Chang and Their Mechanisms

**DOI:** 10.3390/plants13152139

**Published:** 2024-08-01

**Authors:** Jiacheng Wu, Quanzi Bai, Jianghua Chen, Zhenbiao Yang, Xiaoyue Zhu

**Affiliations:** 1 College of Horticulture, Fujian Agriculture and Forestry University, Fuzhou 350002, China; jcwu2940@163.com; 2 Fujian Provincial Key Laboratory of Haixia Applied Plant Systems Biology, Haixia Institute of Science and Technology, School of Future Technology, Fujian Agriculture and Forestry University, Fuzhou 350002, China; 3 CAS Key Laboratory of Tropical Plant Resources and Sustainable Use, Xishuangbanna Tropical Botanical Garden, Chinese Academy of Sciences, Kunming 650223, China; baiquanzi@xtbg.ac.cn (Q.B.); jhchen@xtbg.ac.cn (J.C.); 4 Faculty of Synthetic Biology, Shenzhen University of Advanced Technology, Shenzhen 518055, China; zb.yang1@siat.ac.cn; 5 Key Laboratory of Quantitative Synthetic Biology, Shenzhen Institute of Synthetic Biology, Shenzhen Institute of Advanced Technology, Chinese Academy of Sciences, Shenzhen 518055, China; 6 Haixia Institute of Science and Technology, Fujian Agriculture and Forestry University, Fuzhou 350002, China

**Keywords:** *Camellia* Sect. *Chrysantha* Chang, metabolites, cell senescence, anti-aging natural compounds

## Abstract

Aging is an irreversible pathophysiological process for all organisms. The accumulation of senescent cells in pathological sites or tissues is recognized as the major cause of diseases and disorders during the aging process. Small molecules that reduce senescent cell burdens have gained increasing attention as promising intervention therapeutics against aging, but effective anti-senescence agents remain rare. *Camellia* Sect. *Chrysantha* Chang is documented as a traditional Chinese herbal medicine used by ethnic groups for many medical and health benefits, but its effect on aging is unclear. Here, we investigated the anti-senescence potential of eight *C.* Sect. *Chrysantha* Chang species. The results show that ethyl acetate fractions from these *C.* Sect. *Chrysantha* Chang species were able to delay the senescence of H9c2 cardiomyocytes except for *C*. *pingguoensis* (CPg). N-butanol fractions of *C*. *multipetala* (CM), *C. petelotii* var. *grandiflora* (CPt), and *C*. *longzhouensis* (CL) showed a senescent cell clearance effect by altering the expression levels of senescent-associated marker genes in the DNA-damage response (DDR) pathway and the senescent cell anti-apoptotic pathway (SCAPs). By using UPLC-QTOF-MS-based non-targeted metabolomics analyses, 27 metabolites from Sect. *Chrysantha* species were putatively identified. Among them, high levels of sanchakasaponin C and D in CM, CPt, and CL were recognized as the key bioactive compounds responsible for senescent cell clearance. This study is the first to disclose and compare the anti-cell-senescence effect of a group of *C.* Sect. *Chrysantha* Chang, including some rare species. The combination of senescent markers and metabolomics analyses helped us to reveal the differences in chemical constituents that target senescent cells. Significantly, contrary to the *C. chrysantha* var. *longistyla* (CCL), which is widely cultivated and commercialized for tea drinks, CM, CPt, and CL contain unique chemicals for managing aging and aging-related diseases. The results from this study provide a foundation for species selection in developing small-molecule-based drugs to alleviate diseases and age-related dysfunctions and may potentially be useful for advancing geroscience research.

## 1. Introduction

Aging is a continuous, gradual decrease in physiological function that influences the quality of senior life and longevity. During the aging process, a combination of genetic, environmental, and lifestyle stress factors cause the accumulation of a variety of molecular and cellular damages over time, which, in turn, leads to a progressive deterioration of tissue and cellular functions [1]. In addition, aging represents a primary risk factor for many degenerative pathologies, such as neurodegenerative diseases, metabolic diseases/diabetes, cardiovascular diseases, and cancer [2]. Slowing the biological processes of aging and managing chronic conditions would nurture healthy lives for individuals, reduce the mortality rate for older adults worldwide, and help to meet global healthcare requirements for the aging population from a public health perspective across all countries.

Cellular senescence is an important response to acute or chronic sources of damage, and is featured by cell cycle arrest, i.e., a stable and permanent loss of proliferative capacity [3]. Since the first discovery of senescent cells in prolonged serial subculture of fibroblasts, studies on cellular senescence have attracted more and more attention due to its wide range of important impacts on physiology and pathology, especially in the field of aging. Cellular senescence is essential for embryogenesis, wound healing, and restoring plasticity. It could also act as an anticancer mechanism in our bodies to restrain the proliferation of damaged cells [4]. While spatially and temporally regulated cell senescence is imperative for development and health, studies on dysregulated senescence are growing rapidly due to its detrimental effects on many vital organ systems. Senescent cells are often accompanied by senescence-associated secretory phenotype (SASP), which encompasses a range of cytokines, matrix metalloproteinases, chemokines, etc. The threshold theory postulates that when a certain threshold is passed, SASP could increase the number of senescent cells and induce local and systemic inflammation and tissue disruption [5]. Collectively, disordered senescent cell accumulation and SASP amplification in the microenvironment would result in organ dysfunction. And these processes are implicated in many pathogeneses, including aging-related and chronic diseases [6].

In the last decade, increasing evidence has shown that senescent cell burden is strongly associated with aging. Selective removal of senescent cells can slow down, halt, or reverse aging. Clearing senescent cells from naturally aged mice [7] and a transgenic INK-ATTAC diseased mouse model have been reported to extend the lifespan of mice [8]. Moreover, the depletion of senescent cells with pharmaceuticals has been shown to decrease the incidence and development of diseases and promote health in clinical trials. Although the roles of cell senescence and SASP still remain unambiguous, and a lot of challenges must be overcome in the translation of preclinical cell and animal models to humans, therapeutic approaches to eliminating senescent cells or to suppress SASP hold vast potential for extending human life expectancy and the human healthspan [9].

Cell senescence is driven by complex pathways, and the senescent cells are highly heterogeneous in terms of their underlying molecular mechanisms and physiological functions. No gold standard has been established, and no single marker can fully characterize senescence with high enough sensitivity and specificity, making it difficult to evaluate the outcomes of therapeutic effects. In general, stress signals often cause proliferative arrest through the activation of the p53-Cdkn1a(p21) and/or retinoblastoma (RB)-Cdkn2a(p16) pathways, which then inhibit cycline-dependent kinases (CDKs) and finally induce G1/S cell cycle checkpoint blockade [10]. The expression of transcription factor p53 also governs SASP [11], which feeds back positively or negatively to the senescent cell number depending on the stressors and the spatial–temporal conditions. Diseases and disorders associated with senescent cell accumulation have shown up-regulation in one or more senescent cell anti-apoptotic pathways (SCAPs). Theoretically, cells should undergo apoptosis when they encounter harmful DNA damage and turn on DNA damage response (DDR) pathways [12]. However, some senescent cells could exhibit apoptosis resistance owing to the up-regulation of pro-survival pathways (i.e. SCAPs) and the down-regulation of apoptotic mediators [13]. Moreover, some senescent cells display greater resistance to apoptotic stimuli than non-senescent cells because B-cell lymphoma (*Bcl-2* family) genes, such as anti-apoptosis *Bcl-2*, *Bcl-w*, and *Bcl-xL* and pro-apoptosis *Bax*, are dysregulated in senescent cells [14,15]. Inhibition of the *BcL-2* family through siRNA or small molecules has proven to be effective in clearing senescent cells [16]. Therefore, induction of *BcL-2* family expression is a useful assay for senescence examination. In addition, senescent cells are always featured with lysosomal changes. Hence, the activity of lysosomal senescence-associated beta-galactosidase (SA-β-gal) is also commonly used to reveal cell senescence [17]. To characterize cell senescence more effectively, a combination of markers is suggested, although this may still not be sufficient to address the complexity and kinetics of senescence.

Natural compounds and their derivatives that selectively eliminate senescent cells have emerged as promising therapeutics with which to treat aging-related diseases and disorders. For instance, quercetin, a common flavonoid abundant in numerous plants, has been shown to reduce the mRNA expression of *p16^Ink4a^* and the number of SA-β-gal positive cells in senescence-induced tissues when combined with dasatinib [18]. Oleuropein, the most abundant polyphenol in olives, can protect osteoarthritic chondrocytes from accumulating senescent cells and down-regulate the level of NF-kB and SASP [19]. Prominent in complex structures and diverse scaffolds, natural products are recognized as a treasure trove for developing drug leads. There has been exponential growth in the discovery of drug leads from plants that are both edible and medicinal since they are often pre-validated as harmless and as the key to longevity for centuries. Frequent consumption of edible plants, such as sage and pomegranate, has demonstrated positive effects on our health by boosting the immune system and alleviating inflammation and oxidative stresses [20,21]. The habit of drinking healthy beverages such as tea is associated with reduced risks of developing coronary artery disease and stroke [22]. Additionally, oral administration can improve medication adherence and reduce the risk of hemolysis compared with intravenous injection. But the isolation, screening, and identification of active compounds are challenging. The discovery of nature-product-based anti-aging active compounds from medicinal and food plants will be energized by modern technological developments.

*Camella* Sect. *Chrysantha* (Theaceae), distributed in the southwest of China and Vietnam, is a group of evergreen shrubs famous for their ornamental yellow flowers. Their flowers and leaves have been utilized in Chinese traditional herbal medicine as treatments for dysentery, diarrhea, pharyngitis, and cirrhosis and are documented in traditional medical books such as the *Dictionary of Medicinal Plants* and the *Compendium of Guangxi Ethnic Medicines*. Over 50 species of *C*. Sect. *Chrysantha* Chang have been identified [23]. In recent years, a number of pharmacological studies have revealed that extracts from Sect. *Chrysantha* exhibit anticancer, antioxidation, and anti-inflammation effects [24,25,26]. According to the unitary theory of fundamental aging mechanisms, the pillars of aging are interconnected. Therefore, interventions that target one of the pillars, such as reducing inflammation or oxidative stress, may also alleviate the aging phenotypes [27]. Since most constituents of Sect. *Chrysantha* plants exhibit free radical scavenging activities, theoretically, they might be able to prevent cells from senescence induced by oxidative stress [25]. A recent study revealed that leaf extracts from *C. nitidissima* are rich in phenolic contents and that they showed a significant effect in inhibiting the formation of advanced glycation end products (AGEs), which are associated with the development and progression of many age-related diseases [26]. But few studies have demonstrated whether Sect. Chrysantha could remove senescent cells or compared extracts from different species in terms of their anti-senescence effects, and the pathways that are involved also require further investigation.

A large population of elderly people will develop cardiac injury due to age-dependent stresses. Clinical trials for high-risk populations (such as patients with heart attack risk, participants in the Canakinumab Anti-inflammatory Thrombosis Outcome Study, or those experiencing other cardiovascular events) have garnered significant attention. In this study, we used UPLC-QTOF-MS-based non-targeted metabolomics to analyze active components and profile the association between metabolites and cell senescence. We specifically examined several markers in cell DDR pathways, lysosomal changes, antiapoptotic pro-survival pathways, and the senescent cell burden in accelerated H9c2 cardiomyocyte aging models induced by D-galactose (D-gal). This type of study will lay a foundation from which to unravel the anti-cardiac aging effect of *C.* Sect. *Chrysantha* Chang.

## 2. Results

### 2.1. Anti-Senescence Effect of Eight Species in C. Sect. Chrysantha Chang

**Figure 1:**
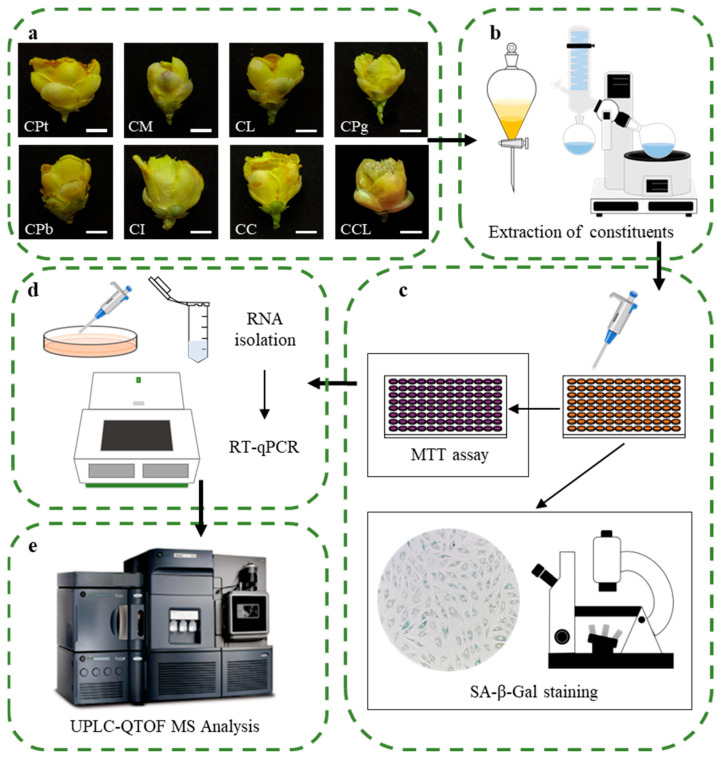
Overview of the workflow for anti-senescence-activity screening of the *C.* Sect. *Chrysantha* Chang chemical constituents. (**a**) Eight species in *C.* Sect. *Chrysantha* Chang: CPt, *C. petelotii* var. *grandiflora*; CM, *C. multipetala*; CL, *C. longzhouensis*; CPg, *C. pingguoensis*; CPb, *C. pubipetala*; CI, *C. impressinervis*; CC, *C. chrysantha* (Hu); CCL, *C. chrysantha* var. *longistyla*. Scale bar = 1 cm. (**b**) Constituents extraction and concentration. (**c**) The anti-senescence activity of extracts assessed via MTT assay and SA-β-Gal staining. (**d**) Extracts altered expression level of genes associated with senescence. (**e**) Identification of chemical constituents.

To evaluate the anti-cell-senescence effect of *C.* Sect. *Chrysantha* Chang, we created an accelerated aging model, followed by constituent extraction, senescence assays, and metabolomic analysis (Figure 1).

**Figure 2:**
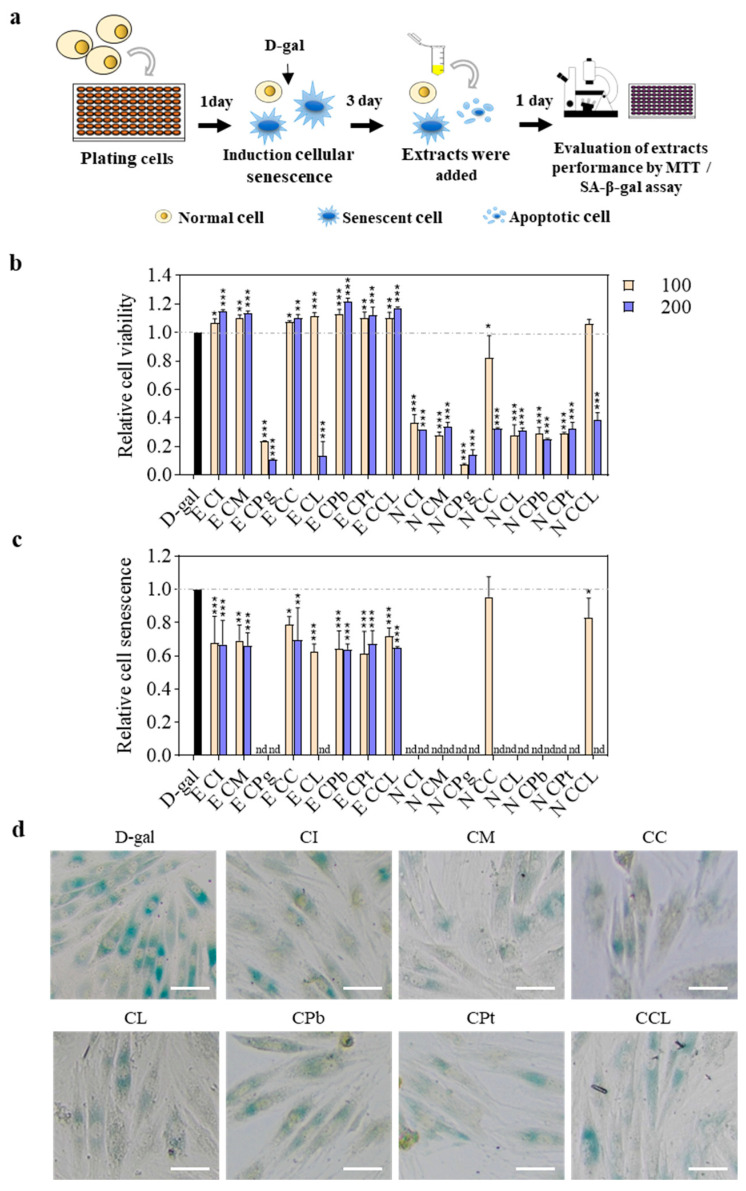
The anti-senescence effect of ethyl acetate and n-butanol extracts of flowers from eight species in *C*. Sect. *Chrysantha* Chang in D-gal-induced senescent cells: (**a**) experimental workflow; (**b**) relative cell viability; (**c**) relative cell senescence; (**d**) cells treated with ethyl acetate extracts at a concentration of 100 μg·mL^−1^ stained for SA-β-Gal. Scale bar = 100 μm. * *p* < 0.05 vs. D-gal, ** *p* < 0.01 vs. D-gal, *** *p* < 0.001 vs. D-gal, nd = not detected, three biological replicates. E, ethyl acetate extract; N, n-butanol extract; CI, *C. impressinervis*; CM, *C. multipetala*; CPg, *C. pingguoensis*; CC, *C. chrysantha* (Hu); CL, *C. longzhouensis*; CPb, *C. pubipetala*; CPt, *C. petelotii* var. *grandiflora*; CCL, *C. chrysantha* var. *longistyla*.

Initially, we focused on examining the effect of Sect. *Chrysantha* extracts on senescent cell burden. D-gal is commonly employed to create stress-induced premature aging models in both cells and animals [28]. D-gal is catalyzed by galactose oxidase to generate aldose and hydroperoxide. An excessive accumulation of these compounds has been shown to lead to inflammation, mitochondrial dysfunction, and aging both *in vivo* and *in vitro* [29]. In this study, H9c2 cells were treated with D-gal to induce accelerated senescence. Subsequently, senescent cells were exposed to ethyl acetate and n-butanol fractions derived from eight species of Sect. *Chrysantha* for 1 d then subjected to an MTT assay and SA-β-gal activity measurement (Figure 2). Relative cell viability and the degree of cell senescence were calculated to establish an accelerated cell aging model for evaluating the effect of different extracts.

**Figure 3:**
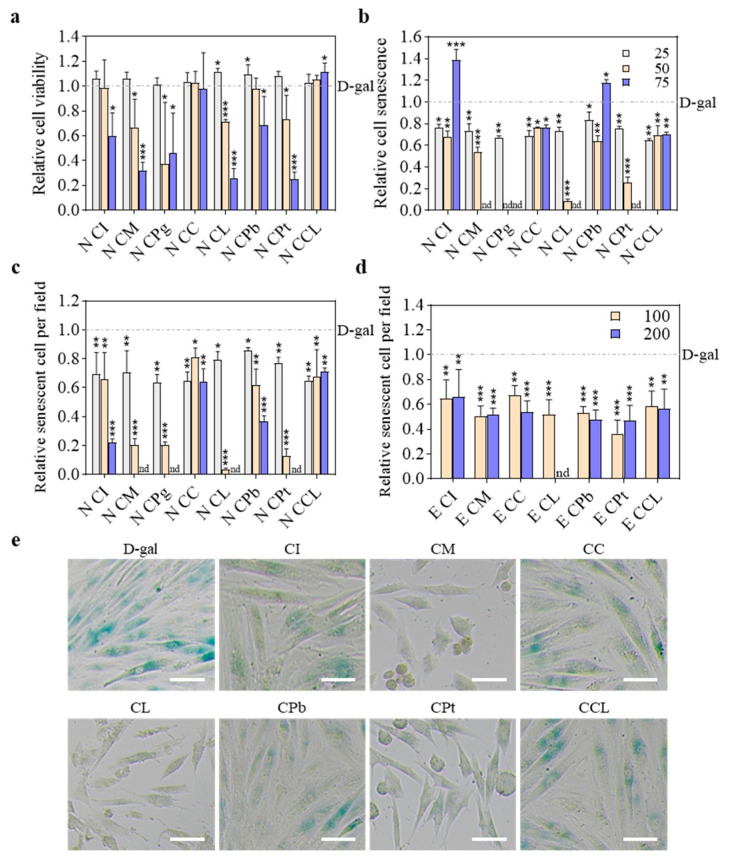
Screening of anti-senescence constituents in extracts from flowers of eight *C*. Sect. *Chrysantha* Chang species in D-gal-induced senescent cells: (**a**) the relative cell viability of n-butanol extracts; (**b**) the relative cell senescence of n-butanol extracts; (**c**) the relative senescent cell number per field of n-butanol extracts; (**d**) the relative senescent cell number per field of ethyl acetate extracts; (**e**) cells treated with n-butanol extracts at the concentration of 50 μg·mL^−1^ stained for SA-β-Gal. Scale bar = 100 μm. * *p* < 0.05 vs. D-gal, ** *p* < 0.01 vs. D-gal, *** *p* < 0.001 vs. D-gal, nd = not detected, three biological replicates. E, ethyl acetate extract; N, n-butanol extract; CI, *C. impressinervis*; CM, *C. multipetala*; CPg, *C. pingguoensis*; CC, *C. chrysantha* (Hu); CL, *C. longzhouensis*; CPb, *C. pubipetala*; CPt, *C. petelotii* var. *grandiflora*; CCL, *C. chrysantha* var. *longistyla*.

The results showed that the ethyl acetate extracts of seven species except for CPg had a significant effect on increasing cell proliferation at concentrations of 100 and 200 μg·mL^−1^ (Figure 2b). Additionally, they significantly reduced the proportion of cell senescence (Figure 2c,d) and their cell counts (Figure 3d), demonstrating a capacity to delay the aging process. Among the n-butanol extracts, CCL exhibited neither cytotoxicity nor a proliferation enhancement effect, although it did reduce the proportion of senescent cells at a concentration of 100 μg·mL^−1^. In contrast, the remaining seven n-butanol extracts displayed significant cytotoxicity at the same concentration (Figure 2b). Once the relative cell viability falls below 0.5, it is very hard to find a sufficient number of cells under microscopy to conduct statistical analysis on relative cell senescence.

Next, the concentration of n-butanol extracts was lowered to a safer range, as many extracts led to cell death at 100 μg·mL^−1^. Among the eight n-butanol extracts, only CM, CL, and CPt markedly reduced cell viability and the proportion and number of senescent cells in each microscope field when 50 μg·mL^−1^ was applied (Figure 3a–c,e), as compared to the untreated senescent control. This result indicated that the reduction in the number of senescent cells was higher than that of proliferative cells. Meanwhile, the n-butanol extracts of CI, CCL, CPb, and CC did not show significant cytotoxicity to senescent cells within the same concentration. On the contrary, CPg induced the death of both proliferative and senescent cells at a concentration of 50 μg·mL^−1^ but did not decrease the viability of senescent cells at a lower concentration. Furthermore, CI, CPb, CCL, and CC failed to reduce the cell viability and proportion of senescent cells at a higher concentration. It is speculated that the n-butanol extracts of CM, CL, and CPt contain high abundances of active constituents capable of specifically eliminating senescent cells.

### 2.2. C. Sect. Chrysantha Chang Extracts Down-Regulate the Expression Level of Bcl-2 Family Genes in Senescent Cells

Senescent cells display differences in gene expression profile, SA-β-gal activity, and apoptosis resistance compared to normal cells [30,31,32]. SA-β-gal activity, *p21^CIPI/WAF1^*, and *p16^INK4a^* are the most commonly used senescence markers. In D-gal-induced aging models, cells have been shown to increase senescence markers, such as senescence-associated genes (*p16^INK4a^*, *p21^CIPI/WAF1^*, *p53*) [33]. Senescent cells express β-galactosidase activity, and the senescence-associated β-galactosidase cleaves X-gal to yield an insoluble blue compound at pH 6.0, which can be detected easily [31].

Due to the complexity of the aging process and chronic diseases, it is important to use complementary tools to test the function of senolytic drugs during drug discovery. It is well established that *p16^INK4a^* and *p21^CIPI/WAF1^* are associated with the abolishment of senescence in preclinical models [5]. In senescent cells, *p53* mediates apoptosis, and the modulation of *p53* is thus commonly used in the first- and second-generation senolytic strategies [34]. Bcl-2 family proteins are the main regulators of intrinsic apoptosis and are involved in cellular senescence. The overexpression of Bcl-2 proteins and the suppression of Bcl-2 associated Bax protein cause apoptosis resistance [35]. *Bcl-2* family genes are typically expressed at higher levels in senescent cells compared to proliferating cells. The *Bax* gene plays a critical role in diseases, including tumorigenesis, autoimmune complications, chronic neurodegenerative conditions, and heart injury. Although Bax dysregulation results in apoptotic dysfunction, and although necrosis is a primary cause of injury during myocardial infarction, reperfusion injury is primarily driven by apoptosis [36]. Given these insights, genetic models such as senescence-linked markers are used, alongside the MTT assay and senescence-associated β-galactosidase activity measurements, to further evaluate the activity of the *C*. Sect. *Chrysantha* extracts.

The above experimental results showed that ethyl acetate and n-butanol extracts from several species in *C*. Sect. *Chrysantha* Chang exhibit notable anti-senescence activities. Interestingly, the low concentration of the n-butanol extracts from CM, CPt, and CL showed characteristics of senescent cell clearance, and most of their ethyl acetate extracts increased cell viability and decreased the proportion of senescent cells. Collectively, we selected n-butanol extracts of CL and CM and ethyl acetate extracts of CL and CPb for further experiments.

**Figure 4:**
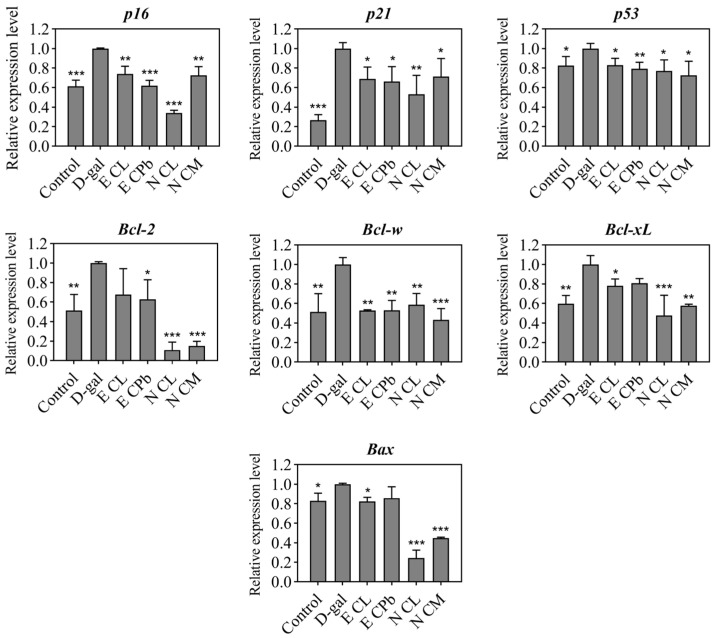
Relative expression levels of cell-senescence-associated genes and *Bcl-2* family genes in D-gal-induced senescent cells. The senescent cells were treated with ethyl acetate extracts of CL and CPb and n-butanol extracts of CL and CM, and gene expression levels of senescence-related genes and apoptosis-associated genes were detected via QRT-PCR. * *p* < 0.05 vs. D-gal, ** *p* < 0.01 vs. D-gal, *** *p* < 0.001 vs. D-gal, three biological replicates. E, ethyl acetate extract; N, n-butanol extract; CL, *C. longzhouensis*; CPb, *C. pubipetala*; CM, *C. multipetala*.

Quantitative RT-PCR results revealed that the expression levels of *p16^INK4a^*, *p21^CIPI/WAF1^*, *p53*, *Bcl-2*, *Bcl-w*, *Bcl-xL*, and *Bax* were up-regulated in the senescence cell group. And the four treatment groups could significantly attenuate the expression levels of *p16^INK4a^*, *p21^CIPI/WAF1^*, and *p53* genes in the senescent group (Figure 4). Both the n-butanol extracts of CL and CM could significantly down-regulate the expression level of three *Bcl-2* family genes and *Bax* genes. No statistically significant difference in *Bcl-2* expression was observed between the CL ethyl acetate extract treatment and the untreated senescent group. Similarly, there were no significant discrepancies in the expression of *Bcl-xL* and *Bax* when comparing the CPb ethyl acetate extract with the untreated senescent group. Despite the fact that the ethyl acetate extracts of CL and CPb have a lesser impact on *Bcl-*2, *Bcl-xL*, and *Bax* genes than the two n-butanol extracts of CL and CM, we postulated that unidentified compounds concentrated in the n-butanol extracts of CL and CM might play a role in the apoptotic processes of senescent cells.

### 2.3. UPLC-QTOF-MS Results Showed That Triterpene Saponins Are Responsible for the Difference in Anti-Senescence Activity between Different Species of C. Sect. Chrysantha Chang

Ultra-high-performance liquid chromatography with quadrupole time-of-flight mass spectrometry (UPLC-QTOF-MS) was employed to provide a comprehensive chemical profiling of the eight *C*. Sect. *Chrysantha* Chang species. After filtering, a total of 279 single-molecular features were included for principal component analysis (PCA). 

**Figure 5:**
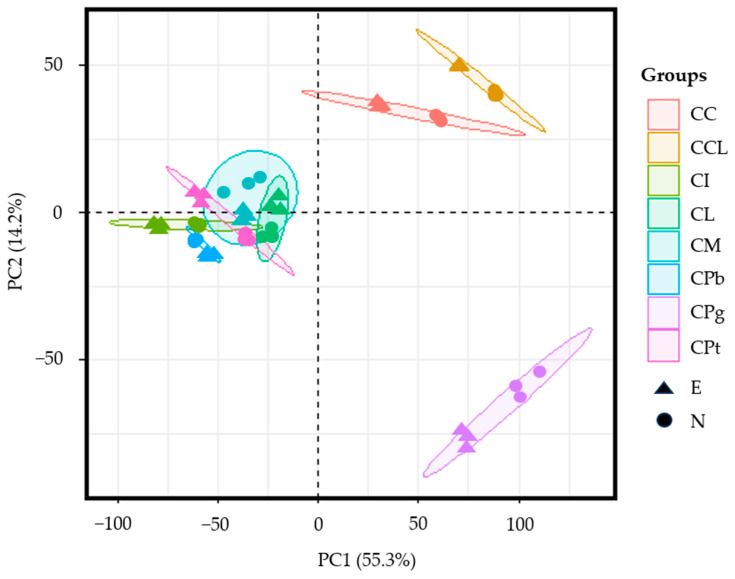
PCA score plot for ethyl acetate and n-butanol extracts of eight different *C*. Sect. *Chrysantha* Chang flowers based on 279 molecular features detected in ESI^-^. E, ethyl acetate extract; N, n-butanol extract; CPg, *C. pingguoensis*; CI, *C. impressinervis*; CPb, *C. pubipetala*; CPt, *C. petelotii* var. *grandiflora*; CL, *C. longzhouensis*; CM, *C. multipetala*; CCL, *C. chrysantha* var. *longistyla*; and CC, *C. chrysantha* (Hu).

In the PCA score plot (Figure 5), the first and the second principal components explained 55.3% and 14.2% of the variation, respectively. The ethyl acetate fraction of each species was close to the n-butanol extraction of the same species. The CI, CPb, CL, CPt, and CM groups were more similar and were distinctly separated from the other three groups (i.e., the CPg, CCL, and CC). Although the PCA analysis results indicated some differences among different species, these differences are caused by species and not by solvents. Further analysis is needed to clarify why the n-butanol extracts of CPt, CL, and CM exhibit anti-cell-senescence activity while their ethyl acetate extracts do not.

Therefore, we screened several substances with high abundance from filtered molecules features and inferred their identities through online databases and literature references. Compounds 1 to 14 were speculated as catechin, epicatechin, procyanidin B2, procyanidin C1, procyanidin tetramer isomer 3 (B type), cinnamtannin A2, quercetin 3-glucoside 7-xyloside, rutin, kaempferol-3-O-glucoside, kaempferol 5-glucoside, quercetin-3-O-glucoside, quercetin-7-O-β-D-glucoside, quercetin 3-arabinoside, and ginsenoside Ro by comparing MS/MS fragmentation patterns and accurate masses from the literature and online databases. Compounds 15 to 27 were putatively identified by comparing the *m*/*z* and formula from the literature related to camellia plants and online databases (Table 1).

**Figure 6:**
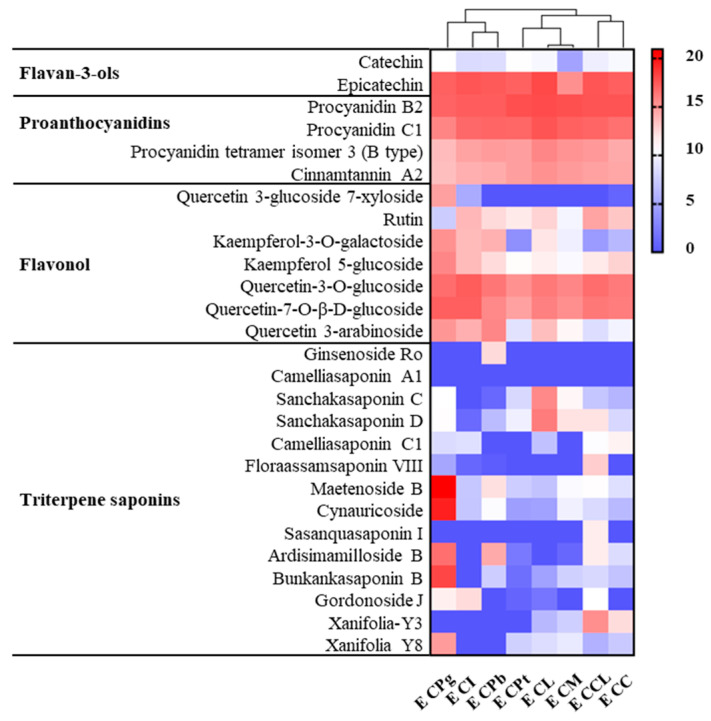
Heat map of compounds’ intensity of ethyl acetate extracts. The relative intensities of the identified compounds in ethyl acetate extracts of different *C*. Sect. *Chrysantha* Chang flowers are demonstrated. E, ethyl acetate extract; CPg, *C. pingguoensis*; CI, *C. impressinervis*; CPb, *C. pubipetala*; CPt, *C. petelotii* var. *grandiflora*; CL, *C. longzhouensis*; CM, *C. multipetala*; CCL, *C. chrysantha* var. *longistyla*; CC, *C. chrysantha* (Hu). This analysis is based on the average signal abundance from three biological replicates for each species. The values expressed as LOG_2_ are shown on a color scale proportional to the content of each metabolite.

**Figure 7:**
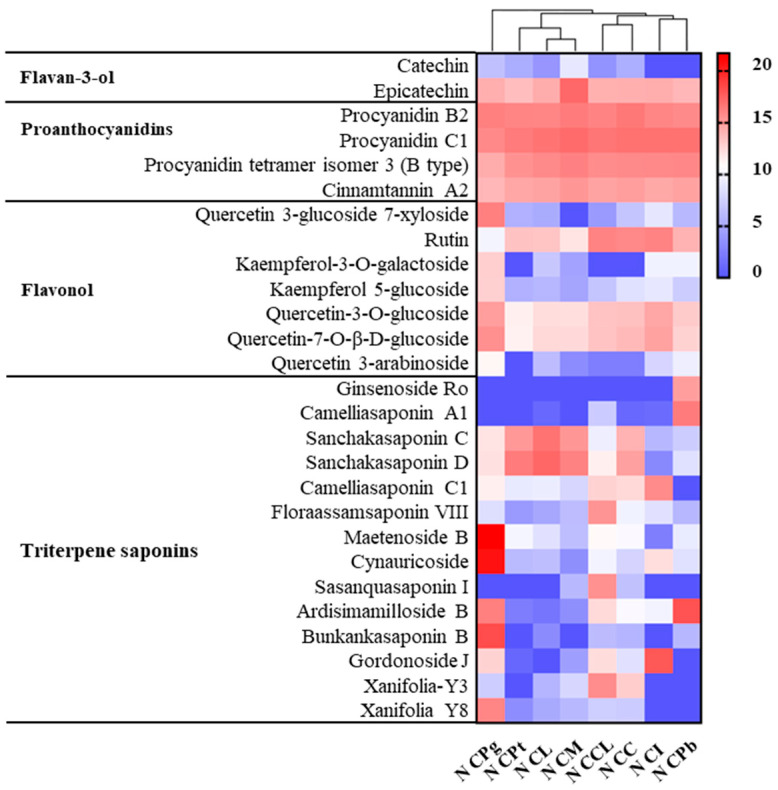
Heat map of compounds’ intensity of n-butanol extracts of eight *C.* Sect. *Chrysantha* Chang flowers. N, n-butanol extract; CPg, *C. pingguoensis*; CPt, *C. petelotii* var. *grandiflora*; CL, *C. longzhouensis*; CM, *C. multipetala;* CCL, *C. chrysantha* var. *longistyla*; CC, *C. chrysantha* (Hu); CI, *C. impressinervis*; CPb, *C. pubipetala*.

Heat maps were plotted to show the relative concentrations of the putatively identified metabolites in ethyl acetate (Figure 6) and n-butanol (Figure 7) extracts. Results showed that there were minor differences in the level of flavan-3-ols, proanthocyanidins, and flavonol among the ethyl acetate extracts of the eight species (Figure 6). Moreover, ginsenosides Ro was uniquely present in CPb. In comparison, sanchakasaponin C and sanchakasaponin D were more abundant in CL, although traces of these compounds were also detected in CPt, CM, and CCL. Camelliasaponin C1 and floraassamsaponin VIII were present in small amounts in CCL and CC. Notably, CPg harbored exceptionally high levels of maetenoside B and cynauricoside, which were scarcely evident in other species. Moreover, CPg exhibited greater concentrations of xanifolia-Y8, ardisimamilloside B, and bunkankasaponin B compared to the other species. These findings indicate that the compositions of ethyl acetate extracts from CPb, CI, CPt, CM, and CL are relatively similar, with the distinguishing factor being the high concentration of saponins in CPg that sets it apart from the rest.

The saponin extracted from these *C*. Sect. *Chrysantha* Chang species were enriched in the n-butanol fraction (Figure 7). Epicatechin, quercetin-7-O-β-D-glucoside, quercetin-3-O-glucoside, and four proanthocyanidins were abundant across all eight species. Quercetin 3-glucoside 7-xyloside, kaempferol-3-O-galactoside, and kaempferol 5-glucoside were relatively high in CPg. And the content of rutin in CPg was lower than that in the other seven species. There were minor differences between the ethyl acetate and n-butanol extracts in terms of the content of flavan-3-ols, proanthocyanidins, and flavonol. Ginsenoside Ro was uniquely presented in CPb, whereas camelliasaponin A1 reached its peak abundance in this species. Sanchakasaponin C and sanchakasaponin D were significantly abundant in CL, CM, and CPt, with less but still notable amounts in CC and CPg. We could also observe that xanifolia-Y8, cynauricoside, maetenoside B, ardisimamilloside B, and bunkankasaponin B were extremely abundant in CPg. Ardisimamilloside B and gordonoside J levels were relatively high in CPb and CC, respectively. The cluster analysis showed that the compositions of CPt, CL, and CM were relatively close. CI, CPb, CCL, and CC were clustered, respectively, while CPg diverged distinctly from the other seven species.

## 3. Discussion

Aging is the biggest biological obstacle in the 21st century. However, aging has not been given a code in the International Classification of Disease (ICD)-11 2024 version, so drug development and clinical trials toward anti-aging interventions need to focus on the prevention or alleviation of age-associated diseases. Among the many targets, senescent cells have become a hotspot because their accumulation within tissues is known to lead to organ dysfunction and aging-associated diseases [3], and reducing their numbers has shown great potential in extending the healthspan in animal models. Cellular senescence can be triggered by either replicative senescence or stress-induced premature senescence [56,57]. An escalated level of ROS within cells is a major factor of oxidative-stress-induced DNA damage [58]. Numerous studies have investigated the anti-oxidation effect of flower or leaf extracts from *C.* Sect. *Chrysantha* Chang [25,59], but they rarely engage with the subject of aging, although oxidative stress is an important stressor in aging. In this work, extracts of CL, CPt, and CM were found to decrease the proportion of oxidative-stress-induced senescent cells, indicating that the extracts of Sect. *Chrysantha* can alleviate oxidative aging through their radical scavenging activities and enhance the lifespan by removing senescent cells.

In the preliminary experiment, it was found that within the established concentration parameters, dichloromethane and water fractions did not exhibit substantial anti-cell-senescence activity. Conversely, the ethyl acetate and n-butanol extracts demonstrated significant anti-senescence effects. Further metabolomics analysis confirmed the enrichment of primary bioactive compounds in the ethyl acetate and n-butanol fractions. Therefore, we focused on looking for bioactive anti-senescence compounds from the ethyl acetate and n-butanol fractions in the subsequent experiments.

Senolytics are a class of drugs that selectively eliminate senescent cells. Natural-product-derived senolytic compounds, such as quercetin [60] and fisetin [61], target cellular senescence through various mechanisms. In this study, 27 compounds were putatively identified based on their accurate masses and MS/MS fragmentation patterns compared via online databases and literature references, including 2 flavan-3-ols, 4 proanthocyanidins, 7 flavonols, and 14 triterpene saponins. The saponins identified in this work include ginsenosides Ro and a group of six saponins isolated from Theaceae species, which have been reported to possess activities relevant to suppressing melanin formation, oxidation [52], tumor growth [55], and allergy [49], respectively. The anti-senescence effect of saponins has been documented for decades. For example, ginsenosides Rg1 can inhibit the formation of advanced glycation end products (AGEs) that are closely linked to aging [62]. We found that the saponins extracted from Sect. *Chrysantha* are more abundant in the n-butanol fraction than in the ethyl acetate fraction. N-butanol extracts from CL, CM, and CPt are more effective at clearing senescent cells compared to those from the other five species. In addition, the metabolite compositional differences between CL, CM, and CPt are minimal. However, the n-butanol extracts contain higher concentrations of two triterpenoid saponins, putatively identified as sanchakasaponin C and sanchakasaponin D. Both have been reported to suppress melanin formation [48]. High contents of sanchakasaponin C and sanchakasaponin D were also detected in the ethyl acetate extract of CL, but the ethyl acetate extracts of CL showed cytotoxicity to cells at a dose of 200 μg·mL^−1^, whereas those of CM and CPt improved cell viability at the same concentration. We hypothesize that cells may die due to the excessive concentrations of sanchakasaponins. We propose that sanchakasaponin C and D are key compounds contributing to the elimination of senescent cells, although further investigation is required to confirm whether they possess senolytic activity.

It has been reported that the expression levels of pro-survival networks are up-regulated in senescent cells, consistent with their resistance to pro-apoptotic stimuli [2]. We found that D-gal treatment elevated the expression levels of *Bcl-2* and *Bax* in senescent H9c2 cells, with a greater degree in *Bcl-2*, reflecting the anti-apoptosis nature of senescent cells. It is known that inhibiting the pro-survival pathway or down-regulating resistance to apoptosis can selectively eliminate the senescent cells, such as with a combination of quercetin and dasatinib [60] and navitoclax, which specifically induces apoptosis in senescent cells by inhibiting Bcl-xL and Bcl-w proteins [63]. Our results showed that the n-butanol extract of CL and CM reduced the transcription levels of *Bcl-2* family genes. Although the expression of the apoptosis regulatory gene *Bax* is also down-regulated, the extent of reduction is less than that of *Bcl-2*, resulting in a lower *Bcl*-*2*/*Bax* ratio that may promote senescent cells apoptosis. However, the ethyl acetate extracts of CL and CPb down-regulate the *Bcl*-*2*/*Bax* ratio to a lesser degree than the n-butanol extracts of CL and CM, thus showing a smaller impact on the viability of senescent cells.

Alongside the proapoptotic, inflammatory, and tissue-disruptive SASP, there are also growth-promoting SASP senescent cells [5]. This study only examined the effect of Sect. *Chrysantha* extracts on oxidative-stress-induced senescent cells. The effect of the extracts on senescent cells induced by different stresses are worth further investigation. Additionally, we have not tested whether these chemicals could inhibit SASP. Furthermore, it is known that intermittent treatment can be as effective as continuous treatment for senolytics in terms of reducing the senescent cell burden [64]. However, we have not tested whether there is any difference in the effects when the chemicals are administered intermittently or continuously. Once the chemicals are purified and a large quantity is obtained, it may be interesting to carry out these experiments.

Moreover, this study marks an initial exploration of the anti-senescent effects of Sect. *C. Chrysantha* extracts. Several markers were chosen to evaluate their impact on senescent cell elimination. Although SA-β-gal staining and the expression of *p16*, *p21*, and other *Bcl-2* family markers are commonly used to identify senescent cells, they could occasionally be expressed in non-senescent stages [17,65]. Because of the heterogeneity of senescent cells and SASP, small molecules that remove senescent myocardiocytes and alter their SASP may not work for other cell types or in different spatial–temporal conditions. It would be beneficial to study the specificity of sanchakasaponin C and D over different types of senescent cells and cell fates (such as non-proliferative quiescent cells, cells in terminal differentiation, and proliferative cancer cells) for the successful development of senolytics. Since senescent cells may exhibit beneficial effects (such as in tissue repair and cancer suppression), it may also be beneficial to determine the optimal timing and conditions for the drugs to overcome adverse effects. Meanwhile, it would also be useful to study the anti-aging effects of the extracts on the dynamics of senescent genes and their modulators.

Aging is characterized by a number of interlinked and fundamental “hallmarks of aging”, such as genomic instability, telomere dysfunction, epigenetic alterations, etc. The geroscience hypothesis is built on the foundation that attenuating the pillars of aging, such as inflammation or oxidative stress, may lead to the alleviation of aging phenotypes. In this study, we showed that Sect. *Chrysantha* extracts may modulate the D-gal-induced cellular senescence, down-regulate the internal oxidative stress caused by high sugar, and decrease senescent cells through preventing or delaying cell phenotypes or attenuating their resistance to apoptosis. We also showed that this is achieved through the down-regulation of the *Bcl-2* family and the relief of oxidative stress. This is the first time a systematic study has been conducted on a group of Sect. Chrysantha species to compare their anti-cell-senescence effects. Although *C. chrysantha* var. *longistyla*., the most widely cultivated and commercialized species for tea drinks, exhibits non-toxic effects, sanchakasaponin C and D were found to be more abundant in CM, CPt, and CL, which points to a new direction for breeding and commercialization. In the future, more research is needed to explore sanchakasaponin’s potential impact on aging.

## 4. Materials and Methods

### 4.1. Materials

The flowers of *C. petelotii* var. *grandiflora*, *C. multipetala*, *C. longzhouensis*, *C. pingguoensis*, *C. pubipetala*, *C. impressinervis*, and *C. chrysantha* (Hu) were collected from Xishuangbanna Tropical Botanical Garden, Chinese Academy of Sciences.

### 4.2. Sample Preparation

Bloomed flowers were collected and freeze-dried (−0.080 mBar, −45 °C). The freeze-dried flowers of *C. chrysantha* var. *longistyla* were purchased from Guangxi Guirentang Co, Ltd. (Guangxi, China). The above sample were coarsely powdered and stored at −80 °C prior to use. Ten grams of the freeze-dried flower powders from each species were extracted with 95% ethanol via ultrasonic extraction three times then evaporated in a rotary evaporator at 35 °C to yield crude ethanol extracts. The ethanol extracts were then dissolved in water and extracted sequentially with dichloromethane, ethyl acetate, and n-butanol three times. The fraction and residual water phase were evaporated at 35 °C to yield the dichloromethane fraction, ethyl acetate fraction, n-butanol fraction, and water fraction, respectively.

### 4.3. Cell Culture

Rat cardiomyocytes cell line H9c2 was purchased from the National Collection of Authenticated Cell Culture (Shanghai, China). H9c2 were cultured in DMEM medium (Gibco/Life Technologies, Waltham, MA, USA) supplemented with 100 units per mL of penicillin, 100 mg·mL^−1^ of streptomycin, and 10% fetal bovine serum in a humidified atmosphere containing 5% CO_2_ at 37 °C. Cells were passaged at a ratio of 1:4 when they reached 80% confluence. Experiments were conducted on cells between passages 3 and 10. In the senescence experiments, H9c2 were plated on 96-well plates at 4500 cells per well one day before senescence induction. To induce cellular senescence, H9c2 cells were cultivated with DMEM medium supplemented with 40g·L^−1^ D-gal for 3 days.

### 4.4. Cell Viability Analysis

H9c2 cell viability was determined via MTT assay. The cell culture media were first replaced with serum-free media containing 0.25 mg·mL^−1^ of MTT. After 4 h, the MTT solution was replaced with 150 µL DMSO. The absorbance value was measured at 570 nm using a microplate reader (Tecan Spark, Männedorf, Switzerland). The relative cell viability is calculated using the following equation:

$$Relative\;cell\;viability=\frac{Mean\;OD_{treatment}-Mean\;OD_{blank}}{Mean\;OD_{D-gal}-Mean\;OD_{blank}}$$

### 4.5. Quantitative Real-Time PCR

Total RNA was extracted using RNApure kit (NG3001S, Hlingene, Shanghai, China) according to the manufacturer’s instructions. For each reaction, 1 µg of total RNA was used to synthesize cDNA with the NovoScript^®^ Plus All-in-one 1st Strand cDNA Synthesis SuperMix (gDNA Purge) (E047-01A, Novoprotein, Shanghai, China). qRT-PCR was performed in a Real-Time PCR system (Bio-Rad CFX96, Hercules, CA, USA) using NovoStart SYBR qPCR SuperMixPlus (E096-01A, Novoprotein). The relative expression was normalized using the expression levels of the GAPDH housekeeping gene as reference. Primer sequences can be found in Table S1.

### 4.6. Senescence-Associated β-Galactosidase (SA-β-gal) Activity

Cells were fixed with 2% paraformaldehyde and 0.2% glutaraldehyde for 10 min at room temperature then washed twice with PBS. The fixed cells were incubated in 100 μL of SA-β-gal staining solution containing 40 mM citric acid/Na_3_PO_4_ buffer, 5 mM K_4_[Fe(CN)_6_] 3H_2_O, 5 mM K_3_[Fe(CN)_6_], 150 mM NaCl, 2 mM MgCl_2_, and 1 mg·mL^−1^ X-gal in distilled water and incubated overnight at 37 °C. Senescent cell were identified via phase contrast microscope (Nikon ECLIPSE Ts2, Tokyo, Japan). The relative cell senescence is calculated using the following equation:

$$\mathrm{Relative}\;\mathrm{cell}\;\mathrm{senescence}\;=\frac{{\left({\mathrm{counts}}_{\mathrm{senescent}\;\mathrm{cells}}÷{\;\mathrm{counts}}_{\mathrm{total}\;\mathrm{cells}}\right)}_{\mathrm{treatment}\;\mathrm{well}}}{{\left({\mathrm{counts}}_{\mathrm{senescent}\;\mathrm{cells}}÷{\;\mathrm{counts}}_{\mathrm{total}\;\mathrm{cells}}\right)}_{D-\mathrm{gal}\;\mathrm{well}}}$$

### 4.7. Metabolite Analysis by UPLC-QTOF MS

Each extract was dissolved in 70% methanol before measurement. Three replicates were analyzed for each sample. Extracts from the eight *C*. Sect. *Chrysantha* Chang species were analyzed on a Waters Acquity UPLC system coupled, in tandem, to a Waters photodiode array (PDA) detector (Waters, Milford, MA, USA) and a SYNAPT G2-Si HDMS QTOF mass spectrometer (Waters, Manchester, UK). Chromatographic separation was performed on a Waters Acquity UPLC HSS T3 column (2.1 × 100 mm, 1.8 µm) at 40 °C. Mobile phase A was 0.1% formic acid of water and mobile phase B was 0.1% formic acid of acetonitrile. The chromatographic elution was 0–2 min (99–93% A), 2–13 min (93–60% A), 13–17 min (60–40% A), and 17–22 min (40–1% A). The flow rate was set at 0.3 mL·min^−1^, and the injection volume was 1 μL. Samples were run in negative ionization mode. The following settings were used: capillary voltage, 1.5 kV; cone voltage, 40 eV; collision energy, 4 eV; source temperature, 120 °C; desolvation temperature, 500 °C; cone gas flow, 50 L/h; desolvation gas flow, 800 L/h; and *m*/*z* range, 50–1200 Da. MS^e^ ramp 10 to 40 eV. The data acquisition mode is set to continuum mode. LockSpray (leucineencephalin) reference ions with *m*/*z* of 554.2615 (for ESI^−^) was infused during data acquisition for online calibration.

### 4.8. Data Processing

Data processing was performed with Progenesis QI software version 2.1 (Nonlinear Dynamics, Newcastle upon Tyne, UK). The steps of peak alignment, peak picking, data normalization and peak assignments were performed using default parameters. The ion forms [M − H]^−^, [M + FA − H]^−^, and [2M − H]^−^ in the negative mode were used to deconvolute the spectral data. The abundance of molecular features and mass features were exported to Excel. The data obtained from Progenesis QI were used for manual peak identification. Identification of metabolites was performed by comparing accurate masses, MS/MS fragmentation patterns, online databases such as COCONUT (https://coconut.naturalproducts.net, accessed on 24 June 2021) and PubChem (https://pubchem.ncbi.nlm.nih.gov/, accessed on 24 June 2021), and literature references. The heat map was generated with online software ClustVis (https://biit.cs.ut.ee/clustvis/, accessed on 5 July 2021). Factoextra R package (version 1.0.7) was used for generating the PCA score plot. The data matrix used for generating the PCA score plot and heat map is listed separately in Tables S2 and S3.

### 4.9. Statistical Analysis

All experiments in this study were performed in biological triplicates, and results were expressed as mean or mean ± standard deviation or average. Statistical analysis of the data was performed via one-way analysis of variance (ANOVA), followed by Duncan’s multiple range test using SPSS statistical software (SPSS version 18.0, SPSS Inc., Chicago, IL, USA). *p* value < 0.05 was considered significant.

## 5. Conclusions

In summary, we explored the anti-cell-senescence properties of eight species in *Camellia* Sect. *Chrysantha* Chang. We found that certain extracts from these species were capable of reducing the burden of senescent cells *in vitro*, suggesting that these extracts might hold promise as anti-aging agents. N-butanol fractions of CM, CPt, and CL demonstrated the ability to clear senescent cells and down-regulate the expression of senescence-associated marker genes and SCAPs pathway genes in the senescent cell anti-apoptotic pathway, indicating that these three fractions can eliminate senescent cells by reducing the resistance of senescent cells to apoptotic stimuli. Metabolomic analysis revealed that sanchakasaponin C and D are among the key active components responsible for this effect. These findings provide new insights into the potential use of plant-derived compounds from *C.* Sect. *Chrysantha* Chang in clearing senescent cells. Once the chemicals are purified and a large quantity is obtained, more research will be carried out to explore sanchakasaponin’s potential impact on tissue and animal aging.

## Figures and Tables

**Table 1:** Metabolites putatively identified from eight species of *C*. Sect. *Chrysantha* Chang by UPLC-QTOF-MS.

	Compound	RT (min)	Detected [M-H]- (*m*/*z*)	Mass Error (ppm)	Formula	MS/MS Fragments	Ref.
	Flavan-3-ols						
1	Catechin	5.37	289.0717	1.7	C_15_H_14_O_6_	245.0593, 203.0553, 123.0556	[37]
2	Epicatechin	6.28	289.0719	2.4	C_15_H_14_O_6_	245.0593, 203.0553, 123.0362	[37]
	Proanthocyanidins						
3	Procyanidin B2	5.8	577.1347	0.2	C_30_H_26_O_12_	451.0663, 425.0531, 407.0410, 289.0455, 125.0159	[38]
4	Procyanidin C1	6.51	865.1964	−1.8	C_45_H_38_O_18_	407.0410, 289.0529, 125.0159	[39]
5	Procyanidin tetramer isomer 3 (B type)	6.73	1153.2601	−1.1	C_60_H_50_O_24_	865.1189, 575.0748, 287.0292	[40]
6	Cinnamtannin A2	7	1153.256	−4.7	C_60_H_50_O_24_	1027.1511, 863.1032, 575.0748, 287.0367	[41]
	Flavonol						
7	Quercetin 3-glucoside 7-xyloside	7.25	595.1306	1.2	C_26_H_28_O_16_	300.0034, 160.8273	[39]
8	Rutin	7.75	609.1456	−1.5	C_27_H_30_O_16_	301.0104, 300.0034	[42]
9	Kaempferol-3-O- galactoside	8.56	447.0928	0.6	C_21_H_20_O_11_	285.0127, 284.0034	[43]
10	Kaempferol 5-glucoside	8.82	447.0928	0.2	C_21_H_20_O_11_	285.0127, 284.0108	[37]
11	Quercetin-3-O- glucoside	7.93	463.0851	0.22	C_21_H_20_O_12_	300.0034, 271.0026, 255.0107	[37]
12	Quercetin-7-O-β-D- glucoside	8.05	463.0856	0	C_21_H_20_O_12_	300.0034, 271.0026, 255.0038	[44]
13	Quercetin 3-arabinoside	8.53	433.0771	0.8	C_20_H_18_O_11_	301.0104, 227.0123	[45]
	Triterpene saponins						
14	Ginsenoside Ro	13.78	955.4879	−2.5	C_48_H_76_O_19_	793.3701	[46]
15	Camelliasaponin A1	13.87	1233.588	−1.9	C_58_H_92_O_25_	1089.4489, 1071.4459	[47]
16	Sanchakasaponin C	16.61	1315.6313	−0.8	C_64_H_100_O_28_	325.1640, 183.0008	[48]
17	Sanchakasaponin D	16.92	1315.6306	−1.3	C_64_H_100_O_28_	325.1640, 183.0008	[48]
18	Camelliasaponin C1	15.46	1203.5771	−2.3	C_58_H_92_O_26_	1023.4171	[49]
19	Floraassamsaponin VIII	14.79	1213.5969	−3	C_60_H_94_O_25_	1141.4485, 1083.4456	[49]
20	Maetenoside B	17.35	1185.6031	−1	C_59_H_94_O_24_	1125.4795	[50]
21	Cynauricoside	16.6	1155.5924	−2.3	C_58_H_92_O_23_	1114.4877	[51]
22	Sasanquasaponin I	11.75	1231.6082	−2.4	C_60_H_96_O_26_	209.9275, 162.8275	[52]
23	Ardisimamilloside B	14.56	1071.5352	0.3	C_53_H_84_O_22_	1069.3882, 1025.4020	[53]
24	Bunkankasaponin B	18.1	1195.5863	−3.1	C_60_H_92_O_24_	1155.495	[53]
25	Gordonoside J	15.77	1173.5665	1	C_57_H_90_O_25_	1147.4326	[54]
26	Xanifolia-Y3	15.6	1125.5454	−2.5	C_56_H_86_O_23_	1099.4331, 1069.4596	[55]
27	Xanifolia Y8	15.71	1139.561	2.7	C_57_H_88_O_23_	325.1562	[55]

## Data Availability

The data presented in this study are included in the article/Supplementary Material.

## Supplementary Materials

The following supporting information can be downloaded at https://www.mdpi.com/article/10.3390/plants13152139/s1: Table S1: Primer sequences; Table S2: Data matrix of PCA score plot; Table S3: Data matrix of heat map.
